# Ionic Porous Aromatic Framework as a Self-Degraded Template for the Synthesis of a Magnetic γ-Fe_2_O_3_/WO_3_·0.5H_2_O Hybrid Nanostructure with Enhanced Photocatalytic Property

**DOI:** 10.3390/molecules26226857

**Published:** 2021-11-13

**Authors:** Man Xu, Kai Wang, Xuan Cao

**Affiliations:** 1 Instrumental Analysis Center, Shenyang University of Chemical Technology, Shenyang 110142, China; xuman.jlu@vip.163.com; 2 College of Science, Shenyang University of Chemical Technology, Shenyang 110142, China; wangkai09181019@163.com

**Keywords:** porous aromatic framework, heterostructure, photocatalytic, ionic porous network

## Abstract

An ionic porous aromatic framework is developed as a self-degraded template to synthesize the magnetic heterostructure of γ-Fe_2_O_3_/WO_3_·0.5H_2_O. The Fe_3_O_4_ polyhedron was obtained with the two-phase method first and then reacted with sodium tungstate to form the γ-Fe_2_O_3_/WO_3_·0.5H_2_O hybrid nanostructure. Under the induction effect of the ionic porous network, the Fe_3_O_4_ phase transformed to the γ-Fe_2_O_3_ state and complexed with WO_3_·0.5H_2_O to form the n-n heterostructure with the n-type WO_3_·0.5H_2_O on the surface of n-type γ-Fe_2_O_3_. Based on a UV-Visible analysis, the magnetic photocatalyst was shown to have a suitable band gap for the catalytic degradation of organic pollutants. Under irradiation, the resulting γ-Fe_2_O_3_/WO_3_·0.5H_2_O sample exhibited a removal efficiency of 95% for RhB in 100 min. The charge transfer mechanism was also studied. After the degradation process, the dispersed powder can be easily separated from the suspension by applying an external magnetic field. The catalytic activity displayed no significant decrease after five recycles. The results present new insights for preparing a hybrid nanostructure photocatalyst and its potential application in harmful pollutant degradation.

## 1. Introduction

The construction of a heterostructure catalyst with different semiconductor constituents has become more popular in recent years [1,2,3,4,5,6]. This combination can improve the efficiency of photocatalytic reaction activity by building an inner electric field to separate charge carriers. It is extremely significant to utilize heterostructure photocatalysts to purify water polluted by dyes because almost 20% of the world’s water pollution is caused by dyes [7,8]. Additionally, heterogeneous photocatalysis shows effectiveness in degrading a wide range of dyes into readily biodegradable compounds and eventually mineralizes them into innocuous carbon dioxide and water [9,10,11,12,13]. Tungsten oxide (WO_3_), with a band gap of 2.5–2.8 eV, is considered to be a possible catalyst due to its suitable response to the solar spectrum in the near ultraviolet and blue regions, stable chemical properties in aqueous solution, good oxidizing ability of the holes in the valence band, high resistance against anodic photo-corrosion, and long-term stability during irradiation [14,15,16]. However, the conduction band (0.5 eV vs. NHE, normal hydrogen electrode) is not negative enough to consume photogenerated electrons for the oxygen reduction, leading to low photocatalytic activity [17]. For the purpose of separating photogenerated electron–hole pairs to improve the catalytic performance, several approaches have been developed in recent years, including surface modification with noble metals, special morphologies/phase control, and the formation of nanomaterial composites [18,19,20]. However, most strategies encounter high costs and uncontrollable morphology with limited improvement of photocatalysis performance, which hinders the practical application of WO_3_-based composites.

γ-Fe_2_O_3_ is a stable form of iron oxide with a cubic structure, which has excellent properties of strong magnetism; it is photocatalytic and has low toxicity and good biocompatibility. It can be widely used in the fields of electromagnetics, biology, water treatment, and medicine [21,22,23,24]. It is a potential semiconductor that can be coupled with WO_3_. In the past decade, efforts have been made to prepare γ-Fe_2_O_3_ under high-temperature pyrolysis [25], sol-gel [26], gas phase deposition [27], combustion synthesis [28], and so on. However, these processes are too complex and require too much energy. In recent years, some researchers have utilized the method of phase transition to prepare compounds because of high raw material utilization and the mild reaction conditions [29,30]. Fe_3_O_4_ is a kind of common and cheap iron oxide, which has almost the same structure as that of γ-Fe_2_O_3_. If we can use an in situ phase transition method to prepare γ-Fe_2_O_3_ from Fe_3_O_4_, it will be cost effective. When the phase transition happens, Fe^2+^ occupies the octahedral B position of cubic Fe_3_O_4_ oxidized to Fe^3+^ [31] The procedure leads to the formation of oxygen vacancy, and so the new cubic γ-Fe_2_O_3_ is not stable. Then, it may need to be connected with another compound such as n-type WO_3_ to form hybrid composites.

Several research works on connecting WO_3_ and Fe_2_O_3_ to form hybrid composites have been reported. Bai et al. synthesized Fe_2_O_3_@WO_3_ by decoration of zero-dimensional (0D) Fe_2_O_3_ nanoparticles on the surface of a three-dimensional (3D) WO_3_ hierarchical framework via an impregnation method [32]. Li et al. reported a novel heterojunction photoanode of a WO_3_@a-Fe_2_O_3_ nanosheet array prepared by multiple steps involving hydrothermal growth, pyrolysis, and calcination [33]. Yin et al. first synthesized two-dimensional WO_3_ nanoplates via a robust intercalation and topochemical conversion route. Then, Fe_2_O_3_ NPs were formed in situ on the surfaces of WO_3_ nanoplates via microwave heating followed by calcination to form hierarchical Fe_2_O_3_@WO_3_ nanostructures [34]. Although these composites showed better relevant chemical properties, the synthetic procedure of these composites is always too complex to be extended to practical application.

Porous aromatic frameworks (PAFs) are emerging functional porous solids, known for their ultra-large surface area (specific surface area > 6000 m^2^ g^−1^), tunable local structure, and high thermal/chemical stability [35,36,37,38]. Due to their tailorable and intrinsic porous structures, densely functionalized PAF samples have huge promise for extreme applications [39,40,41,42,43]. For instance, they can withstand strong acid/alkali, high oxidation, and long-term biological durability, whilst exhibiting the highest selectivity, capacities, and uptake kinetics for the capture of Hg^2+^, Nd^3+^, Cu^2+^, and Pb^2+^ from water [44,45,46,47,48]. In particular, molecularly imprinted PAF solids combining the channel- and specific site-abundant PAFs showed record capacity and kinetics two to three orders of magnitude faster than reported for remarkable polymers for uranium adsorption, which is considered an important developmental milestone in the field of extracting uranium from seawater [47,48,49,50].

In this work, a quaternary pyridinium-type PAF sample (PAF-50) was adopted as a self-degraded template [51,52]. Due to the highly charged network, WO_4_^2−^ anions concentrated around the porous network and γ-Fe_2_O_3_/WO_3_·0.5H_2_O hybrid composites were successfully prepared through a two-step hydrothermal method. This is the first time utilizing phase transition to prepare γ-Fe_2_O_3_/WO_3_·0.5H_2_O with the help of PAF-50. The morphologies and crystalline structure of γ-Fe_2_O_3_/WO_3_·0.5H_2_O were investigated using SEM, TEM, and XRD, respectively. The samples were examined by using UV-Vis diffuse reflectance spectra. Meanwhile, the photocatalytic property of the catalysts was evaluated by decomposing RhB in aqueous solution under visible light irradiation. Remarkably, the γ-Fe_2_O_3_/WO_3_·0.5H_2_O heterojunction was demonstrated to have remarkable photocatalytic characteristics and good reusability compared with that of pure WO_3_.

## 2. Materials and Methods

### 2.1. Chemicals

The chemical and reagents were of analytical grade and used as such without any further purification. All the chemicals viz. sodium tungstate (Na_2_WO_4_·2H_2_O), ferric acetylacetonate (C_15_H_21_FeO_6_), methylbenzene (C_6_H_5_CH_3_), rhodamine B (C_28_H_31_C_1_N_2_O_3_), oleic acid (C_18_H_34_O_2_), hydrazine hydrate (N_2_H_4_·H_2_O), hydrochloric acid (HCl), and absolute ethanol (C_2_H_5_OH) were supplied by Sinopharm Chemical Reagent Co., Ltd. (Shanghai, China). All the solutions were prepared in deionized water obtained from an ultra-filtration system.

### 2.2. Synthesis of Fe_3_O_4_

The synthesis of Fe_3_O_4_ was based on the literature [53] using a low-temperature hydrothermal method in a Teflon-lined stainless steel autoclave with a capacity of 50 mL: 0.035 mol/L ferric acetylacetonate in a mixture of toluene, and oleic acid (volume ratio of 20:10 mL) was added into 10 mL of hydrazine hydrate inside the Teflon-lined stainless steel autoclave to form a two-phase reaction system. The obtained mixed solution was sealed and then kept at 130 °C for 24 h. After reaction, the mixture was poured out by adding ethanol and sonicated with several minutes, washed with ethanol with a magnet to reduce the possibility of impurities in the products, and then dried in air.

### 2.3. Synthesis of γ-Fe_2_O_3_/WO_3_·0.5H_2_O Magnetic Hybrid Nanostructure

Hybrid photocatalysts were fabricated via a hydrothermal method using PAF-50 as a self-degraded template. Briefly, 3 mmol Na_2_WO_4_·2H_2_O and 100 mg PAF-50 (precise type: Cl-PAF-50) were dispersed in 30 mL deionized water; the pH value was adjusted between 4 to 5 by drop-wise addition of concentrated HCl solution under continuous stirring; after adding the Fe_3_O_4_ obtained above, the mixture was transferred to autoclave and maintained at 160 °C for 6 h. The collected sample was rinsed with deionized water and dried in air.

### 2.4. Materials Characterization

XRD (X-ray diffraction) patterns give information on the phase and crystallinity of the as-prepared materials, which were collected on a Rigaku D/Max-2550 diffractometer (Tokyo, Japan) equipped with Cu-Ka radiation (λ = 0.15418 nm) at a scanning range of 20–80° and scanning speed of 5°/min. HRTEM images were obtained using a Tecnai G220S-Twin transmission electron microscope (Hillsboro, OR, USA) at an accelerating voltage of 120 kV, and the images were observed at 200 kV instead of 120 kV. XPS spectra were performed on a Thermo ESCALAB 250 (Waltham, MA, USA) with Al Kα radiation at θ = 90° for the X-ray sources; the binding energies were calibrated using the C 1s peak at 284.8 eV. UV-Visible solid absorbances of the samples were obtained using a PerkinElmer Lambda950 UV-Visible solid spectrometer (Waltham, MA, USA) using BaSO_4_ as a reference. The magnetic properties were investigated with a Quantum Design SQUID-MPMS-XL (San Diego, CA, USA). Magnetic hysteresis loops were measured at 300 K under a magnetic field up to 2 T.

### 2.5. Measurement of Photocatalytic Activity

The photocatalytic activity of the hybrid photocatalysts was evaluated by examining the degradation of Rhodamine B (RhB) in aqueous solution. In a typical reaction procedure, 20 mg of the prepared sample is dispersed into 50 mL dye solution (20 mg/L) and continuously stirred in dark overnight to allow comprehensive adsorption–desorption equilibrium. The sample solutions are irradiated with 500 W xenon lamp, and then withdrawn at regular time intervals followed by separation with a magnet to remove the catalyst. Any change in concentration of RhB was monitored using UV-Vis spectrophotometer during the photoreaction process.

## 3. Results

### 3.1. Surface and Structure Characterization of Samples

**Figure 1:**
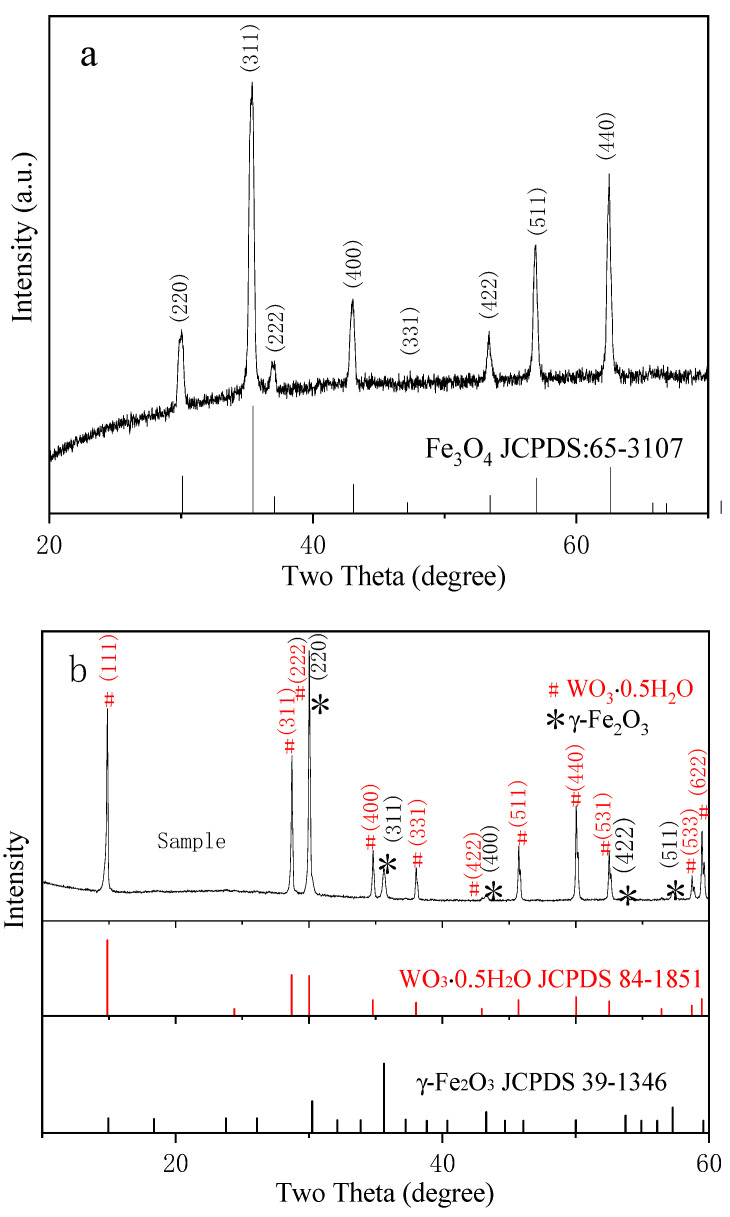

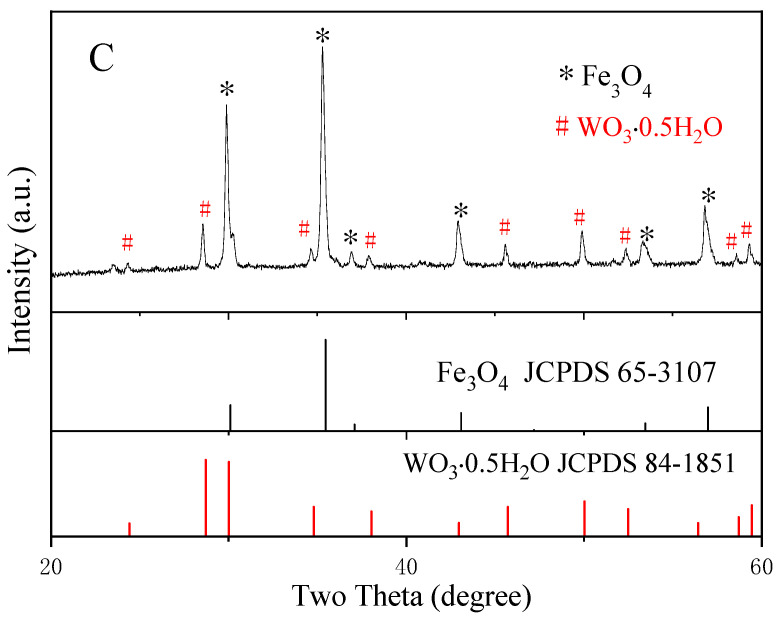
(**a**) Indexed powder XRD pattern of the Fe_3_O_4_ products and ICDD files of Fe_3_O_4_; (**b**) indexed powder XRD pattern of γ-Fe_2_O_3_/WO_3_·0.5H_2_O samples and ICDD files of γ-Fe_2_O_3_ and WO_3_·0.5H_2_O; (**c**) indexed powder XRD pattern of the samples prepared in the absence of PAF-50.

Figure 1 shows the XRD patterns of pure Fe_3_O_4_ and γ-Fe_2_O_3_/WO_3_·0.5H_2_O samples, respectively. As shown in Figure 1a, diffraction peaks (marked as #) are indexed to cubic Fe_3_O_4_ (JCPDS No.: 65-3107). In Figure 1b, the peaks marked with the sample after being loaded with Fe_3_O_4_ show diffraction peaks at (111), (311), (222), (400), (331), (422), (511), (440), (531), (533), and (622), which matched well with WO_3_·0.5H_2_O (JCPDS No.:84-1851), whereas the peaks at (220), (311), (400), (422), and (511) (Marked as *) show diffraction peaks located at γ-Fe_2_O_3_ (JCPDS No.: 39-1346). No additional peaks were observed in the XRD pattern, confirming the purity of the products. In order to explore the effect of template, we prepared γ-Fe_2_O_3_/WO_3_·0.5H_2_O in the absence of PAF-50. The results (Figure 1c) showed that in the products Fe_3_O_4_ did not turn out to be γ-Fe_2_O_3_. We inferred that PAF-50 played a role helping to oxidize Fe_3_O_4_ to γ-Fe_2_O_3_ and in accumulating metal ions to form a complex [26,45,48].

**Figure 2:**
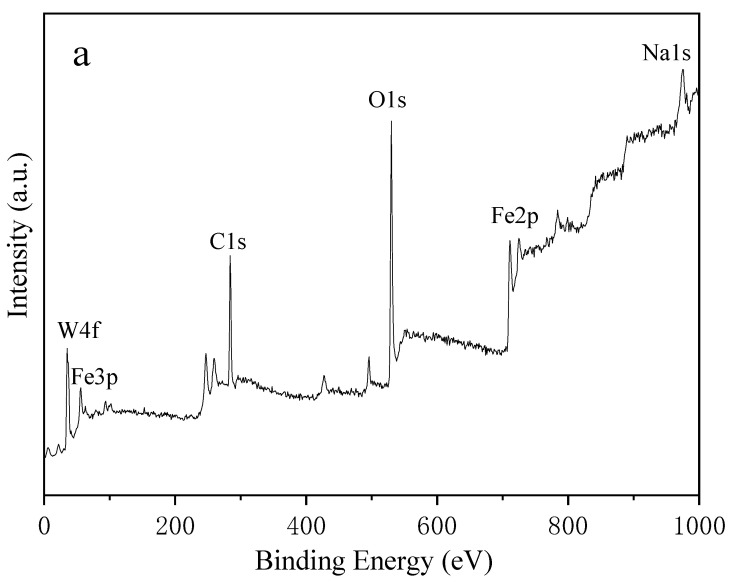

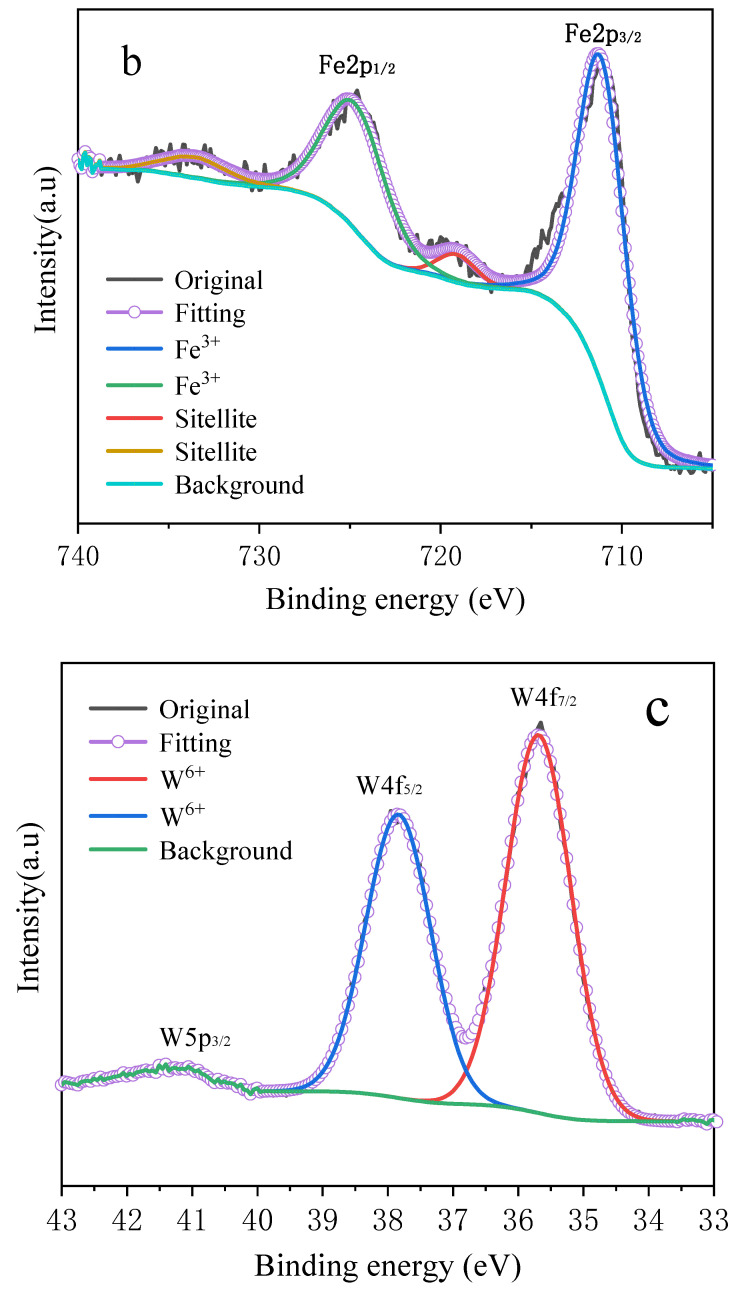
XPS spectra of (**a**) γ-Fe_2_O_3_/WO_3_·0.5H_2_O; (**b**) Fe 2p; (**c**) W 4f.

XPS spectra were performed to determine the valence state of the elements. Figure 2b shows the spectrum of Fe 2p; the locations at 711.1 and 724.8 eV correspond to Fe 2p_3/2_ and Fe 2p_1/2_ [54,55]. The satellite peak of Fe 2p_3/2_ for Fe_2_O_3_ is located approximately 8 eV higher than the main Fe 2p_3/2_ peak. In addition, there appears to be another satellite peak at 733.5 eV, which may be a satellite peak for Fe 2p_1/2_ [56]. The corresponding satellite peak around 719.1 eV confirms the γ-Fe_2_O_3_ phase, which is consistent with the results of XRD [57]. During this hydrothermal reaction, Fe_3_O_4_ has a phase transformation to Fe_2_O_3_. In Figure 2c, the W 4f binding energies of two peaks located at 35.7 eV and 37.8 eV correspond to +6 valence of W. The whole spectra (Figure 2a) show that content of W is much higher than that of Fe. We can infer that WO_3_ covers the surface of Fe_x_O_y_ during the synthesis of hybrid nanostructure. Additionally, XPS is a surface-sensitive analytical technique [58]. Thus, the intensity of Fe 2p peak is weak.

**Figure 3:**
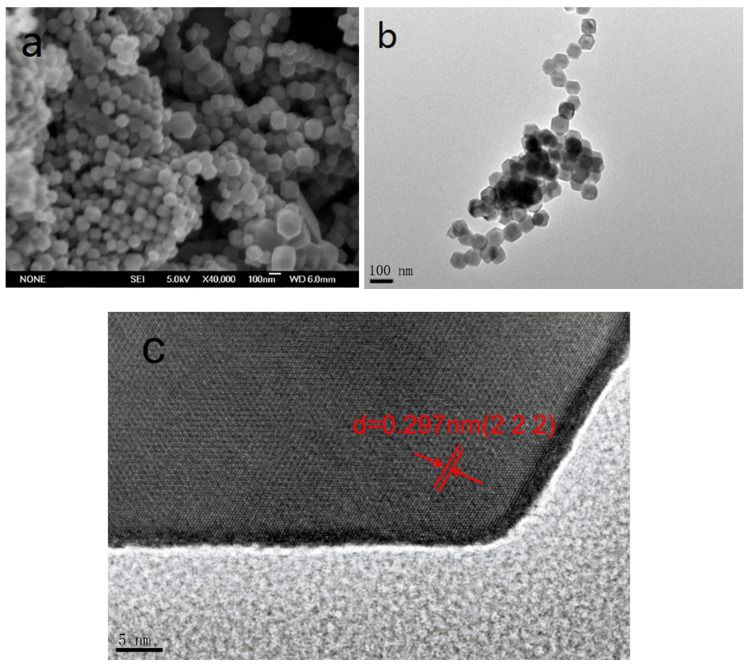
(**a**) SEM image of Fe_3_O_4_; (**b**) TEM image of γ-Fe_2_O_3_/WO_3_·0.5H_2_O; (**c**) HRTEM image of a single particle of γ-Fe_2_O_3_/WO_3_·0.5H_2_O.

Figure 3a,b show the representative images of Fe_3_O_4_ and γ-Fe_2_O_3_/WO_3_·0.5H_2_O. It is evident from Figure 3a that Fe_3_O_4_ shows uniform and regular twelve faces with a size range from 80 to 100 nm. There is no significant change after composed with tungsten. A single particle was selected, and the relevant HRTEM image is shown in Figure 3c. The edge coupled with the center presents a strong contrast, which further proves the formation of well-defined products. There is more than one lattice overlap in the polyhedron, with a lattice spacing of 0.297 nm corresponding to WO_3_·0.5H_2_O (JCPDS No.: 84-1851). In addition, the obvious lattice spacing in the HRTEM image further confirms that the sample comprises two phases of γ-Fe_2_O_3_ and WO_3_ to form a hybrid structure, and the morphology has no significant change after composition.

### 3.2. Magnetism Measurement

**Figure 4:**
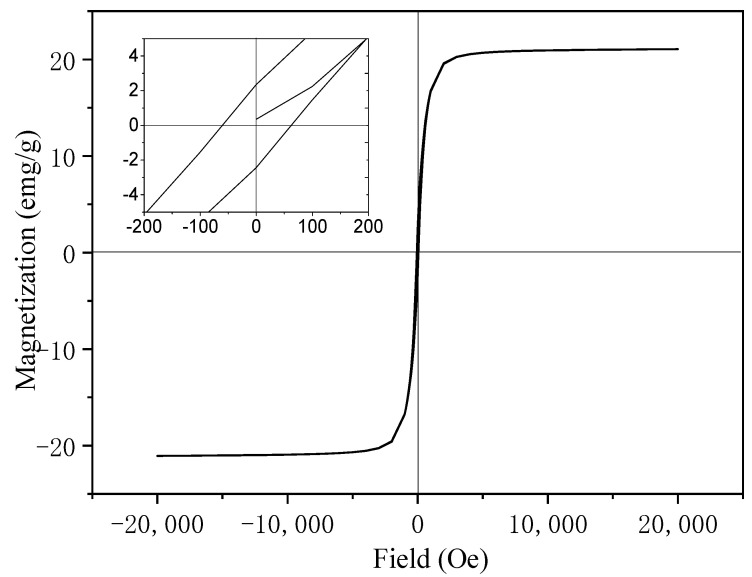
Magnetization hysteresis (M-H) loops measured at 300 K (the inset is a partially enlarged image of the curve).

Figure 4 shows the hysteresis loops of γ-Fe_2_O_3_/WO_3_·0.5H_2_O at 300 K with the applied field ±2T. The saturation magnetization of the heterostructure increases with the field and tends to slow until ±2000 Oe. From the enlarged part of the illustration, we can see that the product is ferromagnetic with a residual magnetization (Mr) of 2.3 emu/g and a coercivity (Hc) of 61.2 Oe [59]. The saturation magnetization (Ms) of 20 emu/g is less than that of the bulk γ-Fe_2_O_3_ of 73.5 emu/g [60]. This is mainly due to the surface of the hybrid structure being covered with hydrated tungsten oxide, which affects the morphology and the spin density, which in turn affects the saturation magnetization.

### 3.3. Photocatalytic Activity

**Figure 5:**
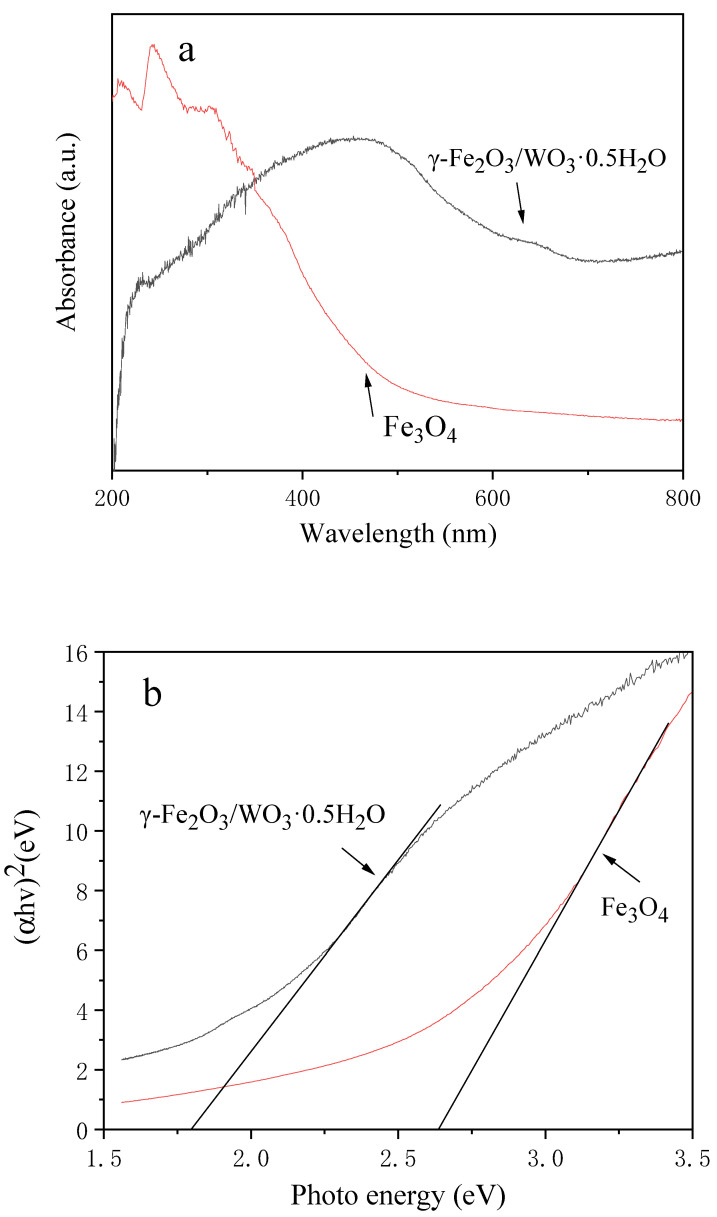
(**a**) UV-Vis diffuse reflectance spectrum of Fe_3_O_4_ and γ-Fe_2_O_3_/WO_3_·0.5H_2_O; (**b**) (αhν)^2^ versus hv curves of Fe_3_O_4_ and γ-Fe_2_O_3_/WO_3_·0.5H_2_O.

Both WO_3_ and γ-Fe_2_O_3_ are typical n-type semiconductors, and the n-n-type heterostructure has absorption in the ultraviolet region and visible region [61]. UV-Visible spectrum analysis was used to explore the light response. As shown in Figure 5a, compared with Fe_3_O_4_, the complex can absorb light in a larger wavelength range. The adsorption band is at 300 to 600 nm, which can absorb ultraviolet and visible light. The light density of the xenon lamp and solar light density is 120 mW/cm^2^ and 54 mW/cm^2^, respectively; therefore, the 500 W xenon lamp was used as the light source in this experiment [62]. The band gap of γ-Fe_2_O_3_/WO_3_·0.5H_2_O can be obtained from the plot of (αhν)^2^ versus hν by extrapolating the strait portion of (αhν)^2^ to zero, as shown in Figure 5b. After calculation, the value was determined to be 1.8 eV, which is narrower than 2.64 eV of Fe_3_O_4_. A narrow band gap is beneficial for the efficient utilization of visible light for photocatalysis [63].

**Figure 6:**
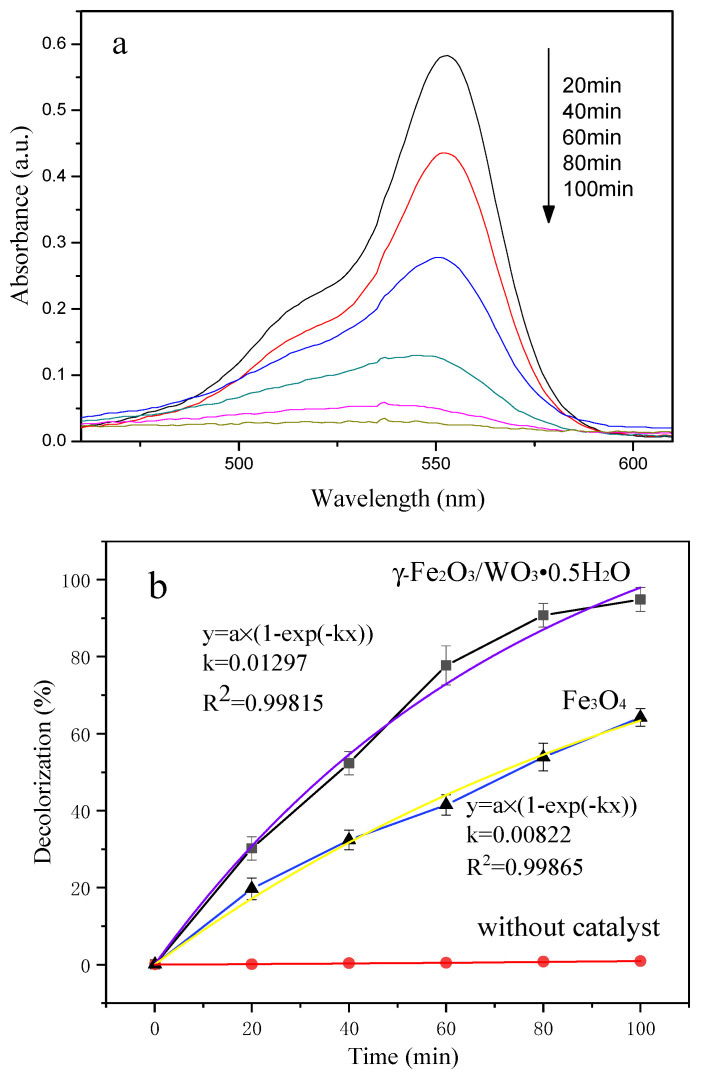
(**a**) Absorption spectrum of RhB solution in the presence of γ-Fe_2_O_3_/WO_3_·0.5H_2_O under UV-Visible light; (**b**) degrading efficiency at different times with various or without catalysts under UV-Visible light. The purple curve and yellow curve are the pseudo-first-order models’ fitting curve.

Rhodamine B (RhB) was used to simulate pollutants in water under UV-Visible light at room temperature. γ-Fe_2_O_3_/WO_3_·0.5H_2_O was dispersed, and the absorption spectrum of the solution was tested. The curve of absorbance wavelength versus time is shown in Figure 6a. The intensity of the absorption peak at 553 nm [64] gradually decreased with the increase in time, and the absorption peak was blue shifted, which indicated that the ethyl on RHB molecule was removed. The characteristic absorption peak of Rhodamine B could hardly be seen at 100 min, and Rhodamine B was completely degraded.

From Figure 6b, the photocatalytic reactions over both Fe_3_O_4_ and γ-Fe_2_O_3_/WO_3_·0.5H_2_O obey first-order kinetics. The degradation percentages of RhB only reached 62% and 1% after photodegradation for 100 min under UV irradiation in the case of the presence of pure Fe_3_O_4_ and in the absence of any catalysts, respectively, which are too low compared to that of γ-Fe_2_O_3_/WO_3_·0.5H_2_O heterostructures (94.9%), revealing the significantly enhanced photocatalytic properties of the γ-Fe_2_O_3_/WO_3_·0.5H_2_O heterostructures. This removal efficiency is more than that of pure WO_3_ [65] or γ-Fe_2_O_3_ [66] according to relevant studies. However, γ-Fe_2_O_3_/WO_3_·0.5H_2_O takes more time than Fe_2_O_3_/WO_3_ in the degradation of RhB with the same concentration [32].

The composite catalyst is the key factor for this degradation reaction, which is mainly due to the synergistic and coupling effects of Fe_2_O_3_ and WO_3_. When γ-Fe_2_O_3_ excited by visible ultraviolet, the electron flow to the valence band of WO_3_, and the hole in the valence band migrates from WO_3_ to γ-Fe_2_O_3_. At the same time, the internal electric field also promotes the electron hole migration, and the formation of the heterostructure promotes the separation of photo-generated electrons and hole pairs [67]. This can reduce the recombination probability and reduce the energy needed for the transition, so it has a better photocatalytic effect than single WO_3_ or γ-Fe_2_O_3_. However, the lower removal efficiency as compared to the reported Fe_2_O_3_/WO_3_ may due to the water molecules in the structure of γ-Fe_2_O_3_/WO_3_·0.5H_2_O.

**Figure 7:**
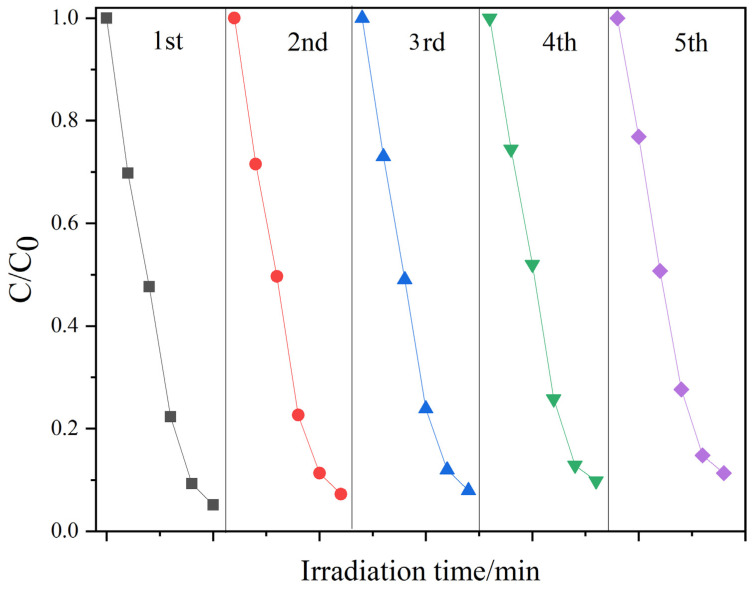
Recyclability of RhB photodegradation with γ-Fe_2_O_3_/WO_3_·0.5H_2_O.

The catalytic activities of the complex were measured by the photodegradation of RhB for five recycles. After each cycle, fresh RhB solution was used for the next photocatalytic experiment. Additionally, the photocatalyst was collected from the previous experiment followed by washing and drying. It is shown in Figure 7 that the catalytic activity displayed no significant decrease after five recycles with a period of 100 min. This indicates that the stability of γ-Fe_2_O_3_/WO_3_·0.5H_2_O is excellent, and it can keep its high photocatalytic activity after the photocatalytic reaction and recycling process [32,68].

**Figure 8:**
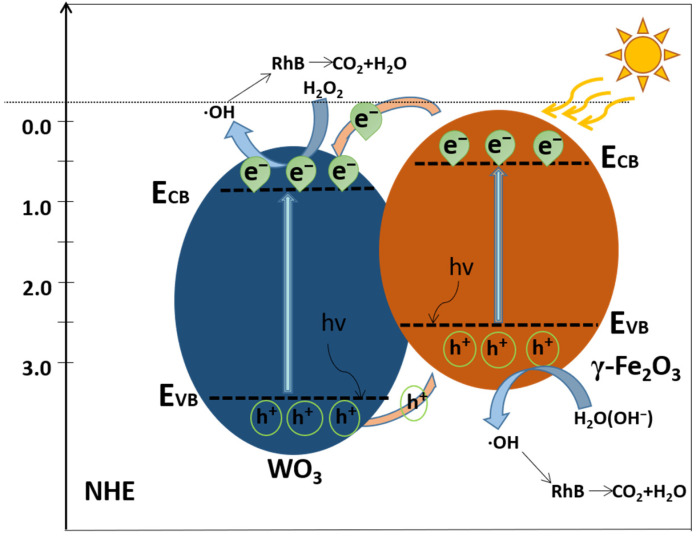
A possible mechanism for the charge-transfer mechanism in γ-Fe_2_O_3_/WO_3_·0.5H_2_O.

In conventional photocatalysts, the photoinduced electrons and holes migrate randomly, and the recombination of the charge carriers reduces the quantum yield in the catalytic process. We can see in Figure 8 that when γ-Fe_2_O_3_ (as the primary light absorber) forms a heterojunction with WO_3_ (as an electron acceptor), the band bending formed at the interface between γ-Fe_2_O_3_ and WO_3_ impels the carriers to diffuse in opposite directions until their Fermi levels reach equivalence [69]. As other studies have claimed that the CB edge potential of γ-Fe_2_O_3_ (0.29 eV) is lower than that of WO_3_ (0.79 eV) [32,70,71], upon irradiation, the ground-state γ-Fe_2_O_3_ and WO_3_ go to an exited state to produce some electron–hole pairs because of their narrow band gaps. Therefore, the photo-excited electrons on the CB of γ-Fe_2_O_3_ transferred to the CB of WO_3_. Additionally, the VB edge potential of WO_3_ (2.60 eV) was found to be larger than that of the γ-Fe_2_O_3_ (2.20 eV), which helps the photo-excited holes on the VB of WO_3_ transfer to the VB of γ-Fe_2_O_3_. The electrons and holes transfer rapidly in the opposite direction due to the heterojunction-generated inner electric field [72]. In this regard, the recombination rate of charge carriers is remarkably reduced, and the γ-Fe_2_O_3_/WO_3_·0.5H_2_O shows enhanced photoactivity for organic pollution degradation under light.

## 4. Conclusions

In conclusion, γ-Fe_2_O_3_/WO_3_·0.5H_2_O heterostructures were synthesized using an ionic porous aromatic framework as self-degraded template and a facile low-temperature hydrothermal growth method. The experimental results show that the structures are composed of γ-Fe_3_O_4_ and WO_3_. The role of PAF-50 is to accumulate metal ion and oxidize Fe_3_O_4_ to Fe_2_O_3_. Compared with Fe_3_O_4_, the complex can absorb light in a larger wavelength range. The results of the degradation experiment revealed that product loading with WO_3_ exhibited higher photocatalytic activity than with pure Fe_3_O_4_, and the degradation efficiency reached 95% for the RhB solution after 100 min. After the degradation process, the dispersed powder can be easily separated from the suspension by applying an external magnetic field. The charge transfer mechanism in γ-Fe_2_O_3_/WO_3_·0.5H_2_O shows that the enhanced photocatalytic properties of the heterostructures are attributed to the larger spectral range, a narrower band gap, and a lower recombination rate of electrons and holes.

## Data Availability

The data presented in this study are available on request from the corresponding author.

## Sample Availability

Samples of the compounds in this study are available from the authors.

## References

[1] Wang H., Zhang L., Chen Z. (2014). Semiconductor heterojunction photocatalysts: Design, construction, and photocatalytic performances. Chem. Soc. Rev..

[2] Qu Y., Duan X. (2013). Progress, challenge and perspective of heterogeneous photocatalysts. Chem. Soc. Rev..

[3] Li K., Han M., Chen R. (2016). Hexagonal@ Cubic CdS Core@ Shell Nanorod Photocatalyst for Highly Active Production of H_2_ with Unprecedented Stability. Adv. Mater..

[4] Yadav A.A., Kang S.W., Hunge Y.M. (2021). Photocatalytic degradation of Rhodamine B using graphitic carbon nitride photocatalyst. J. Mater. Sci. Mater. Electron..

[5] Hunge Y.M., Yadav A.A., Khan S., Takagi K. (2021). Photocatalytic degradation of bisphenol A using titanium dioxide@nanodiamond composites under UV light illumination. J. Colloid Interface Sci..

[6] Hunge Y.M., Yadav A.A., Kang S.W. (2022). Photocatalytic degradation of tetracycline antibiotics using hydrothermally synthesized two-dimensional molybdenum disulfide/titanium dioxide composites. J. Colloid Interface Sci..

[7] Forgacs E., Cserháti T., Oros G. (2004). Removal of synthetic dyes from wastewaters: A review. Environ. Int..

[8] Clarke E.A., Anliker R. (1980). Organic Dyes and Pigments.

[9] Mashentseva A.A., Barsbay M., Aimanova N.A., Zdorovets M.V. (2021). Application of silver-loaded composite track-etched membranes for photocatalytic decomposition of methylene blue under visible light. Membranes.

[10] Parale V.G., Kim T., Lee K.Y., Phadtare V.D., Dhavale R.P., Jung H. (2020). Hydrophobic TiO_2_–SiO_2_ composite aerogels synthesized via in situ epoxy-ring opening polymerization and sol-gel process for enhanced degradation activity. Ceram. Int..

[11] Vdp A., Vgp A., Tk A., Kyl A., Ank B., Hc A. (2021). Ultrasonically dispersed ultrathin g-C_3_N_4_ nanosheet/BaBi_2_Nb_2_O_9_ heterojunction photocatalysts for efficient photocatalytic degradation of organic pollutant. J. Alloy. Compd..

[12] Kim T., Parale V., Jung H.N.R., Kim Y., Driss Z., Driss D. (2019). Facile synthesis of SnO_2_ aerogel/reduced graphene oxide nanocomposites via in situ annealing for the photocatalytic degradation of methyl orange. Nanomaterials.

[13] Parale V.G., Kim T., Phadtare V.D., Han W., Park H.H. (2019). SnO_2_ aerogel deposited onto polymer-derived carbon foam for environmental remediation. J. Mol. Liq..

[14] Liu S., Zhang F., Li H. (2012). Acetone detection properties of single crystalline tungsten oxide plates synthesized by hydrothermal method using cetyltrimethyl ammonium bromide supermolecular template. Sens. Actuators B Chem..

[15] Jiao Z., Wang J., Ke L. (2011). Morphology-tailored synthesis of tungsten trioxide (hydrate) thin films and their photocatalytic properties. ACS Appl. Mater. Interfaces.

[16] Santato C., Ulmann M., Augustynski J. (2001). Photoelectrochemical properties of nanostructured tungsten trioxide films. J. Phys. Chem. B..

[17] Seifollahi Bazarjani M., Hojamberdiev M., Morita K. (2013). Visible light photocatalysis with c-WO_3–x_/WO_3_ × H_2_O nanoheterostructures in situ formed in mesoporous polycarbosilane-siloxane polymer. J. Am. Chem. Soc..

[18] Liu Z., Zhao Z.G., Miyauchi M. (2009). Efficient visible light active CaFe_2_O_4_/WO_3_ based composite photocatalysts: Effect of interfacial modification. J. Phys. Chem. C.

[19] Zhang L.J., Li S., Liu B.K. (2014). Highly efficient CdS/WO_3_ photocatalysts: Z-scheme photocatalytic mechanism for their enhanced photocatalytic H_2_ evolution under visible light. ACS Catal..

[20] Cao J., Luo B., Lin H. (2012). Thermodecomposition synthesis of WO_3_/H_2_WO_4_ heterostructures with enhanced visible light photocatalytic properties. Appl. Catal. B Environ..

[21] Cao S.W., Zhu Y.J., Ma M.Y. (2008). Hierarchically nanostructured magnetic hollow spheres of Fe_3_O_4_ and gamma-Fe_2_O_3_: Preparation and potential application in drug delivery. J. Phys. Chem. C.

[22] Kamali S., Yu E., Bates B. (2020). Magnetic properties of γ-Fe_2_O_3_ nanoparticles in a porous SiO_2_ shell for drug delivery. J. Phys. Condens. Matter.

[23] Sheikholeslami Z., Kebria D.Y., Qaderi F. (2019). Application of γ-Fe_2_O_3_ nanoparticles for pollution removal from water with visible light. J. Mol. Liq..

[24] Feng J., Shi Q., Li Y. (2019). Pyrolysis preparation of poly-γ-glutamic acid derived amorphous carbon nitride for supporting Ag and γ-Fe_2_O_3_ nanocomposites with catalytic and antibacterial activity. Mater. Sci. Eng. C.

[25] Asuha S., Zhao Y.M., Zhao S. (2012). Synthesis of mesoporous maghemite with high surface area and its adsorptive properties. Solid State Sci..

[26] Kyoungja W., Lee H.J. (2004). Synthesis and magnetism of hematite and maghemite nanoparticles. J. Magn. Magn. Mater..

[27] Bomatí-Miguel O., Mazeina L., Navrotsky A. (2008). Calorimetric study of maghemite nanoparticles synthesized by laser-induced pyrolysis. Chem. Mater..

[28] Zhao N., Ma W., Cui Z., Song W., Xu C., Gao M. (2009). Polyhedral maghemite nanocrystals prepared by a flame synthetic method: Preparations, characterizations, and catalytic properties. ACS Nano.

[29] Gang Z., Wang T., Shao Y. (2017). A novel mild phase-transition to prepare black phosphorus nanosheets with excellent energy applications. Small.

[30] Almeida T.P., Fay M., Zhu Y., Brown P.D. (2009). Process map for the hydrothermal synthesis of α-Fe_2_O_3_ nanorods. J. Phys. Chem. C.

[31] Kojima H., Hanada K. (1980). Origin of coercivity changes during the oxidation of Fe_3_O_4_ to γ-Fe_2_O_3_. IEEE Trans. Magn..

[32] Bai S.L., Zhang K.W., Sun J.H. (2014). Surface decoration of WO_3_ architectures with Fe_2_O_3_ nanoparticles for visible-light-driven photocatalysis. Crystengcomm.

[33] Li Y., Zhang L.H., Liu R.R., Cao Z., Sun X.M. (2016). WO_3_@α-Fe_2_O_3_ heterojunction arrays with improved photoelectrochemical behavior for neutral ph water splitting. ChemCatChem.

[34] Yin L., Chen D., Feng M., Ge L., Yang D., Song Z. (2015). Hierarchical Fe_2_O_3_@WO_3_ nanostructures with ultrahigh specific surface areas: Microwave-assisted synthesis and enhanced h2s-sensing performance. RSC Adv..

[35] Ben T., Ren H., Ma S., Cao D., Lan J., Jing X., Wang W., Xu J., Deng F., Simmons J.M. (2009). Targeted synthesis of a porous aromatic framework with high stability and exceptionally high surface area. Angew. Chem. Int. Ed..

[36] Yuan Y., Zhu G. (2019). Porous aromatic frameworks as a platform for multifunctional applications. ACS Cent. Sci..

[37] Yuan Y., Yuan Y., Zhu G. (2020). Multifunctional porous aromatic frameworks: State of the art and opportunities. EnergyChem.

[38] Yuan Y., Yuan Y., Zhu G. (2020). Molecularly Imprinted Porous Aromatic Frameworks for Molecular Recognition. ACS Cent. Sci..

[39] Yang Y., Deng D., Zhang S., Meng Q., Li Z., Wang Z., Sha H., Faller R., Bian Z., Zou X. (2020). Porous organic frameworks featured by distinct confining fields for the selective hydrogenation of biomass-derived ketones. Adv. Mater..

[40] Yan Z., Yuan Y., Tian Y., Zhang D., Zhu G. (2015). Highly efficient enrichment of volatile iodine by charged porous aromatic frameworks with three sorption sites. Angew. Chem. Int. Ed..

[41] Yuan Y., Yang Y., Faheem M., Zou X., Ma X., Wang Z., Meng Q., Wang L., Zhao S., Zhu G. (2018). Molecularly imprinted porous aromatic frameworks serving as porous artificial enzymes. Adv. Mater..

[42] Meng Q., Huang Y., Deng D., Yang Y., Sha H., Zou X., Faller R., Yuan Y., Zhu G. (2020). Porous Aromatic Framework Nanosheets Anchored with Lewis Pairs for Efficient and Recyclable Heterogeneous Catalysis. Adv. Sci..

[43] Yang Y., Faheem M., Wang L., Meng Q., Sha H., Yang N., Yuan Y., Zhu G. (2018). Surface pore engineering of covalent organic frameworks for ammonia capture through synergistic multivariate and open metal site approaches. ACS Cent. Sci..

[44] Yuan Y., Cui P., Tian Y., Zou X., Zhou Y., Sun F., Zhu G. (2016). Cou-pling fullerene into porous aromatic frameworks for gas selective sorption. Chem. Sci..

[45] Yang Y., Yan Z., Wang L., Meng Q., Yuan Y., Zhu G. (2018). Con-structing synergistic groups in porous aromatic frameworks for the selective removal and recovery of Lead(II) ions. J. Mater. Chem. A.

[46] Demir S., Brune N.K., Van Humbeck J.F. (2016). Extraction of Lanthanide and Actinide Ions from Aqueous Mixtures Using a Carboxylic Acid-Functionalized Porous Aromatic Framework. Acs Cent..

[47] Yuan Y., Meng Q., Faheem M., Yang Y., Li Z., Wang Z., Deng D., Sun F., He H., Huang Y. (2019). A molecular coordination template strategy for designing selective porous aromatic framework materials for uranyl capture. ACS Cent. Sci..

[48] Li Z., Meng Q., Yang Y., Zou X., Yuan Y., Zhu G. (2020). Constructing amidoxime-modified porous adsorbents with open architecture for cost-effective and efficient uranium extraction. Chem. Sci..

[49] Li Z., Zhu G., Yuan Y. (2019). Preparation of phosphoric acid based porous aromatic framework for uranium extraction. Acta Chim. Sin..

[50] Wang Z., Meng Q., Ma R., Wang Z., Yang Y., Sha H., Ma X., Ruan X., Zou X., Yuan Y. (2020). Constructing an ion pathway for uranium extraction from seawater. Chem.

[51] Yuan Y., Sun F., Zhang F., Ren H., Guo M., Cai K., Jing X., Gao X., Zhu G. (2013). Targeted synthesis of porous aromatic frame- works and their composites for versatile, facile, efficacious, and durable antibacterial polymer coatings. Adv. Mater..

[52] Yuan Y., Sun F., Li L., Cui P., Zhu G. (2014). Porous aromatic frame-works with anion-templated pore apertures serving as polymeric sieves. Nat. Commun..

[53] Cheng X.L., Jiang J.S., Jiang D.M. (2014). Synthesis of rhombic dodecahedral Fe_3_O_4_ nanocrystals with exposed high-energy {110} facets and their peroxidase-like activity and lithium storage properties. J. Phys. Chem. C.

[54] Tahir A.A., Wijayantha K.G.U., Saremi-Yarahmadi S. (2009). Nanostructured alpha-Fe_2_O_3_ thin films for photoelectrochemical hydrogen generation. Chem. Mater..

[55] Yang S., Wang C., Ma L. (2013). Substitution of WO_3_ in V_2_O_5_/WO_3_-TiO_2_ by Fe_2_O_3_ for selective catalytic reduction of NO with NH_3_. Catal. Sci. Technol..

[56] Yamashita T., Hayes P. (2008). Analysis of XPS spectra of Fe^2+^ and Fe^3+^ ions in oxide materials. Appl. Surf. Sci..

[57] Coey J.M.D., Khalafalla D. (1972). Superparamagnetic γ-Fe_2_O_3_. Phys. Status Solidi A.

[58] Dombrowski K.E., Wright S.E., Birkbeck J.C. (1996). Surface Analysis of Proteins and Related Molecules by X-ray Photoelectron Spectroscopy (XPS). FASEB J..

[59] Mou F., Guan J., Xiao Z. (2011). Solvent-mediated synthesis of magnetic Fe_2_O_3_ chestnut-like amorphous-core/gamma-phase-shell hierarchical nanostructures with strong As(V) removal capability. J. Mater. Chem..

[60] Lu A.H., Salabas E.L., Schueth F. (2007). Magnetic nanoparticles: Synthesis, protection, functionalization, and application. Angew. Chem. Int. Ed..

[61] Veeralingam S., Badhulika S. (2021). Bi-Metallic sulphides 1D Bi_2_S_3_ microneedles/1D RuS_2_ nano-rods based n-n heterojunction for large area, flexible and high-performance broadband photodetector. J. Alloy. Compd..

[62] Hou W.C., Chowdhury I., Goodwin D.G. (2015). Photochemical Transformation of Graphene Oxide in Sunlight. Environ. Sci. Technol..

[63] Wang W., Li N., Chi Y., Li Y.J., Yan W.F. (2013). Electrospinning of magnetical bismuth ferrite nanofibers with photocatalytic activity. Ceram. Int..

[64] Yao T., Cui T., Wang H. (2014). A simple way to prepare Au@polypyrrole/Fe_3_O_4_ hollow capsules with high stability and their application in catalytic reduction of methylene blue dye. Nanoscale.

[65] Xiao Z.L., Wu X.Y., Tan H.Y., Paolo A., Hao S.Y. (2021). Effect of Zn on Photocatalytic Activity of Block-Shaped Monoclinic WO_3_. Chin. J. Inorg. Chem..

[66] Fatimah I., Fadhilah S., Mawardani S.A. (2018). γ-Fe_2_O_3_ Nanoparticles immobilized in SiO_2_ aerogel synthesized from rice husk ash for photofenton like degradation of rhodamine B. Rasayan J. Chem..

[67] Chen J., Wei X., Zhang R. (2021). Type-II C_2_N/ZnTe Van Der Waals Heterostructure: A Promising Photocatalyst for Water Splitting. Adv. Mater. Interfaces.

[68] Guo J.X., Zhou X.Y., Lu Y.B. (2012). Monodisperse spindle-like FeWO_4_ nanoparticles: Controlled hydrothermal synthesis and enhanced optical properties. J. Solid State Chem..

[69] Bai S., Yang X., Liu C. (2018). An integrating photoanode of WO_3_/Fe_2_O_3_ heterojunction decorated with NiFe-LDH to improve PEC water splitting efficiency. ACS Sustain. Chem. Eng..

[70] Senthil R.A., Theerthagiri J., Selvi A. (2017). Synthesis and characterization of low-cost g-C_3_N_4_/TiO_2_ composite with enhanced photocatalytic performance under visible-light irradiation. Opt. Mater..

[71] Ranjbar M., Taher M.A., Sam A. (2015). NiO nanostructures: Novel solvent-less solid-state synthesis, characterization and MB photocatalytic degradation. J. Mater. Sci. Mater. Electron..

[72] Theerthagiri J., Senthil R.A., Priya A., Madhavan J. (2014). Photocatalytic and photoelectrochemical studies of visible-light active α-Fe_2_O_3_–g-C_3_N_4_ nanocomposites. RSC Adv..

